# Higher Triglyceride–Glucose Index Is Associated With Increased Likelihood of Kidney Stones

**DOI:** 10.3389/fendo.2021.774567

**Published:** 2021-11-29

**Authors:** Zheng Qin, Junjie Zhao, Jiwen Geng, Kaixi Chang, Ruoxi Liao, Baihai Su

**Affiliations:** ^1^ Department of Nephrology, National Clinical Research Center for Geriatrics, West China Hospital, Sichuan University, Chengdu, China; ^2^ Med+ Biomaterial Institute of West China Hospital/West China School of Medicine of Sichuan University, Chengdu, China; ^3^ Med-X Center for Materials, Sichuan University, Chengdu, China; ^4^ West China School of Medicine, West China Hospital of Sichuan University, Chengdu, China

**Keywords:** insulin resistance, triglyceride–glucose index, kidney stones, NHANES, cross-sectional study

## Abstract

**Aims:**

We aimed to assess the association between triglyceride–glucose (TyG) index and kidney stones in US adults.

**Methods:**

Data were obtained from the 2007–2014 National Health and Nutrition Examination Survey (NHANES). Participants aged ≥18 years who were not pregnant and provided complete data about TyG index and kidney stones were included in the analysis. Weighted multivariable regression analysis and subgroup analysis were preformed to estimate the independent relationship between TyG index and nephrolithiasis and recurrence.

**Results:**

A total of 20,972 participants were included with the mean TyG index of 8.71 ± 0.72. The prevalence rates of nephrolithiasis and recurrence were 9.30% and 3.17% overall and increased with the higher TyG index tertiles (Nephrolithiasis: Tertile 1, 6.98%; Tertile 2, 9.15%; Tertile 3, 11.98%, p < 0.01; Recurrence: Tertile 1, 1.84%; Tertile 2, 3.27%; Tertile 3, 4.50%, p < 0.01). Each unit increase in TyG index was associated with 12% and 26% higher odds of nephrolithiasis [odds ratio (OR) = 1.12; 95% CI: 1.02–1.22; p = 0.02] and recurrence (OR = 1.26; 95% CI: 1.08–1.46; p < 0.01). Interaction tests indicated no significant effect of gender, age, body mass index, hypertension, and diabetes on this association between TyG index and kidney stones.

**Conclusions:**

Higher TyG index was associated with an increased likelihood of nephrolithiasis and recurrence. Considering TyG index is a reliable indicator of insulin resistance (IR). Treatment and management of IR at a younger age may improve or alleviate the occurrence and recurrence of kidney stones.

## Introduction

Nephrolithiasis is caused by the abnormal accumulation of crystalline substances in the kidney and represents a significant economic and public health burden worldwide (1). The prevalence of nephrolithiasis was estimated to be 7.2%–7.7% globally and showed an increasing tendency with years, with 5%–10% in Europe, 4% in South America, and 1%–19% in Asia, respectively (2, 3). Nephrolithiasis also shows a high recurrence rate of approximately 50% at 10 years (4). In addition, a graded relation was found between kidney stones and the increasing risk of kidney function loss, even end-stage renal disease (5).

Triglyceride–glucose (TyG) index is a logarithmized product of fasting triglyceride and fasting glucose. It has been regarded as a novel and reliable indicator of insulin resistance (IR), a forerunner of type 2 diabetes (6). TyG index is more convenient and easily accessible in clinical practice compared with the plasma insulin in the homeostasis model assessment of IR (7). Previous studies have demonstrated that the elevation of TyG index correlates well with several diseases such as arterial stiffness, coronary artery stenosis, and erectile dysfunction (8–10).

Accumulating evidence showed that IR may increase the risk of nephrolithiasis. Riese and Sakhaee (11) found that formation of uric acid kidney stones appeared to stem on the background of IR, which decreases urinary pH. Hamm (12) reported that IR could also lead to a defect in renal acid excretion and then cause hypocitraturia, which is a significant risk factor for calcium stones. Ando et al. (13) also reported that metabolic syndrome components could increase the risk of kidney stones through IR and subclinical hyperinsulinemia for Japanese women. In addition, increased IR could lower urinary citrate excretion and increase urinary calcium excretion, thus contributing to the formation of calcium stones (14). Since TyG index has been proposed as a marker of IR, it can be speculated that there might be a relationship between TyG index and kidney stones. However, no previous study has assessed the association between TyG index and kidney stones before.

Hence, we explored the association between the TyG index and the likelihood of kidney stones using the 2007–2014 National Health and Nutrition Examination Survey (NHANES) data regarding US men. We hypothesized that higher TyG index was associated with an increased likelihood of kidney stones.

## Materials and Methods

### Study Population

We obtained data from NHANES, a study aimed to evaluate the health and nutrition status of the US population administered by the National Center for Health Statistics (NCHS). The included samples in NHANES have good representativeness because of the stratified multistage probability sampling method adopted in the study design. All NHANES data are publicly available at https://www.cdc.gov/nchs/nhanes/.

**Figure 1:**
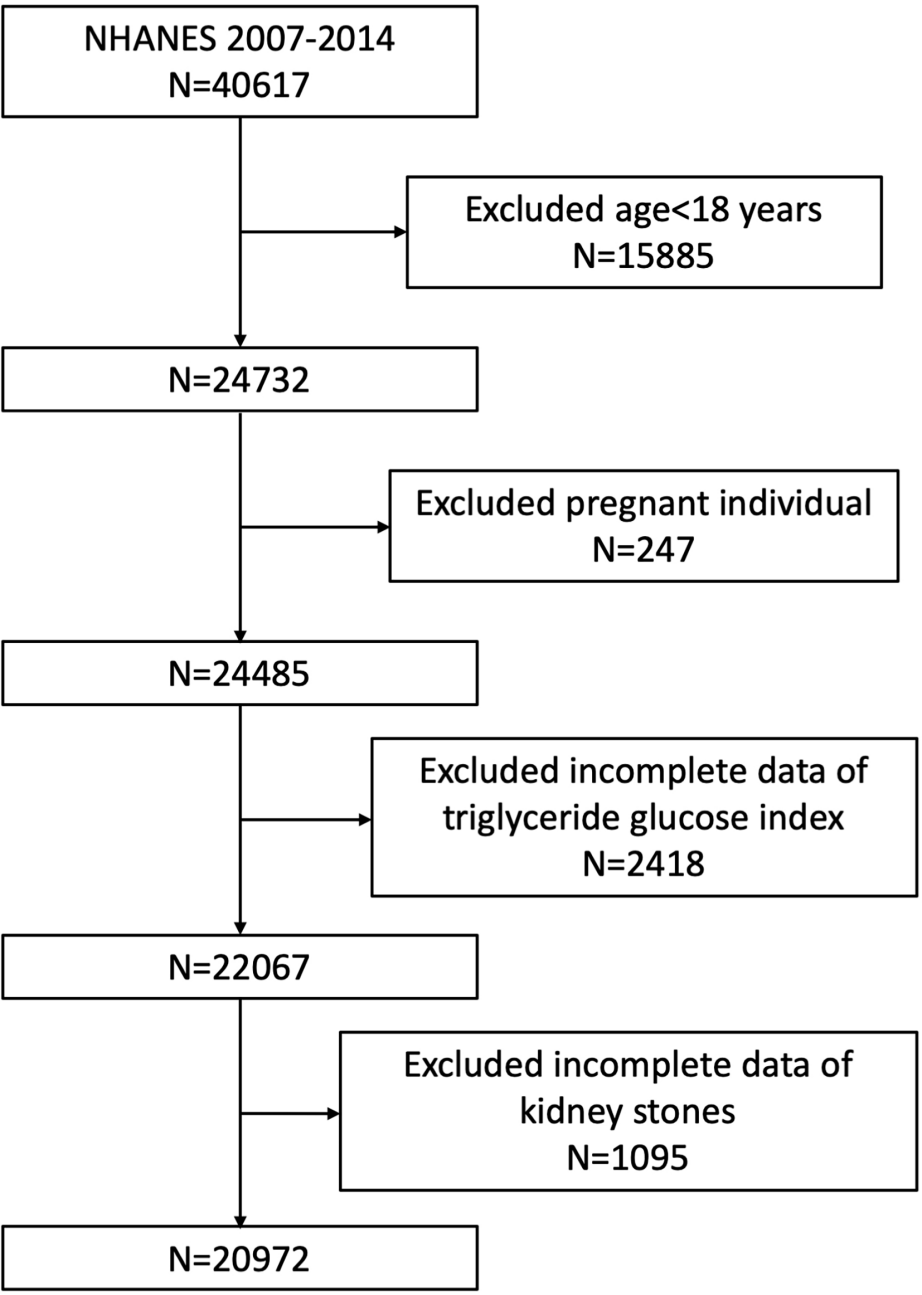
Flowchart of the sample selection from National Health and Nutrition Examination Survey (NHANES) 2007–2014.

Our study was based on four NHANES survey cycles from 2007 to 2014, since only these four cycles include data on both kidney stones (including recurrence of passing kidney stones) and TyG index. A total of 40,617 participants were enrolled at first, after the exclusion of individuals younger than 18 years (n = 15,885), pregnant (n = 247), missing the data on TyG index (n = 2,418), and missing the data on kidney stones (n = 1,095), 20,972 participants were included in our final analysis **(** **Figure 1** **)**.

The NCHS Research Ethics Review Board granted the human subject approval for the conduction of NHANES, and written informed consent was obtained from each participant.

### Exposure and Outcome Definitions

TyG was designed as an exposure variable. We calculated TyG index as Ln [triglycerides (mg/dl) * fasting glucose (mg/dl)/2] (15). Both the concentrations of triglycerides and fasting glucose were measured by an enzymatic assay using automatic biochemistry analyzer. Serum triglyceride concentration was measured using the Roche Modular P and Roche Cobas 6000 chemistry analyzers. Fasting plasma glucose was measured by the hexokinase-mediated reaction using Roche/Hitachi Cobas C 501 chemistry analyzer.

Two questions about kidney conditions were used to assess the kidney stones, including “Have you/Has sample person (SP) ever has a kidney stone?” and “How many times have you/has SP passes a kidney stone?” Previous studies have validated the accuracy of self-reported kidney stone conditions (16). If a participant answered yes to ever had a kidney stone, he was considered to have nephrolithiasis. An individual who has experienced two or more times of having kidney stones was considered to have a recurrence of passing kidney stones. Both the occurrence of nephrolithiasis and nephrolithiasis recurrence were designed as outcome variables.

### Covariates

Potential covariates that might confound the association between TyG index and kidney stones were summarized in the multivariable-adjusted models. Covariates in our study included gender (male/female), age (years), race, education level, poverty-to-income ratio (PIR), marital status (married or living with a partner/single), alcohol intake (never/up to once a week/2–3 times a week/4–6 times a week/daily of more), physical activity (vigorous/moderate/less than moderate), cholesterol level (mg/dl), body mass index (BMI), smoking status (smoking or not), hypertension, and diabetes. BMI was categorized as <25, 25–29.9, and ≥30 kg/m^2^, which corresponded to normal weight, overweight, and obese population for all participants. All detailed measurement processes of study variables were publicly available at www.cdc.gov/nchs/nhanes/.

### Statistical Analysis

All statistical analyses were conducted according to Centers for Disease Control and Prevention (CDC) guidelines, and an appropriate NHANES sampling weight was applied and accounted for complex multistage cluster survey design in the analysis. Continuous variables were presented as mean with standard deviation, and categorical variables were presented as a percentage. Either a weighted Student’s t-test (for continuous variables) or weighted chi-square test (for categorical variables) was used to evaluate the differences in groups divided by TyG index (tertiles). Multivariate logistic regression models were employed to explore the independent relationship between TyG index and kidney stones in three different models. In model 1, no covariates were adjusted. Model 2 was adjusted for gender, age, and race. Model 3 was adjusted for gender, age, race, education level, poverty-to-income ratio, marital status, alcohol intake, physical activity, cholesterol, BMI, smoking status, hypertension, and diabetes. Smooth curve fitting (penalized spline method) and weighted generalized additive model (GAM) regression were conducted to further assess the nonlinear relationship between TyG index and kidney stones. Subgroup analysis stratified by gender, age, BMI, hypertension, and diabetes was also performed by stratified multivariate regression analysis. In addition, an interaction term was added to test the heterogeneity of associations between the subgroups using log likelihood ratio test model. p < 0.05 was considered statistically significant. All analyses were preformed using Empower software (www.empowerstats.com; X&Y solutions, Inc., Boston, MA, USA) and R version 3.4.3 (http://www.R-project.org, The R Foundation).

## Results

### Baseline Characteristics of Participants

Weighted demographic baseline characteristics of included participants were shown in **Table 1**. A total of 20,972 participants were included in our study, of whom 48.86% were male and 51.54% were female, with the average age of 47.42 ± 16.77 years. The mean of TyG index was 8.71 ± 0.72, and the ranges of TyG index for tertiles 1–3 were 5.87–8.37, 8.38–8.99, and 8.99–13.21, respectively. The average prevalence of nephrolithiasis was 9.30% overall; 6.98%, 9.15%, and 11.98% for Tertile 1, Tertile 2, and Tertile 3. The prevalence of nephrolithiasis recurrence was 3.17% for the whole participants, and participants in higher TyG tertile trended to have higher rates of nephrolithiasis recurrence (Tertile 1, 1.84%; Tertile 2, 3.27%; Tertile 3, 4.50%, p < 0.01).

**Table 1:** Baseline characteristics of participants, weighted.

	Overall (5.87–13.21)	Tertile 1 (5.87–8.37)	Tertile 2 (8.38–8.99)	Tertile 3 (8.99–13.21)	p value
Age (years)	47.42 ± 16.77	42.89 ± 16.52	48.39 ± 16.95	51.29 ± 15.67	<0.01
Gender (%)					
Male	48.86	40.47	48.77	58.05	<0.01
Female	51.14	59.53	51.23	41.95	
Race (%)					
Mexican American	8.46	6.53	8.56	10.45	<0.01
Other Hispanic	5.50	5.39	5.21	5.91	
Non-Hispanic White	68.33	65.32	70.16	69.64	
Non-Hispanic Black	10.59	15.72	9.26	6.47	
Other Races	7.12	7.05	6.82	7.54	
Marital status (%)					
Married or with partner	63.20	58.96	64.37	66.57	<0.01
Single	36.80	41.04	35.63	33.43	
Education level (%)					
Less than high school	17.61	13.95	18.01	21.15	<0.01
High school or GED	22.38	20.29	22.19	24.84	
Above high school	59.93	65.70	59.74	53.88	
Unknown	0.08	0.05	0.06	0.13	
RIP (%)					
≤1	15.42	15.62	15.13	15.50	0.71
>1	84.58	84.38	84.87	84.50	
BMI category^1^ (%)					
Normal weight	31.07	48.69	28.88	14.24	<0.01
Overweight	33.64	30.51	35.93	34.62	
Obese	35.29	20.81	35.19	51.14	
Alcohol intake per week (%)					
Never	17.42	13.67	17.01	21.86	<0.01
Up to once a week	51.49	52.84	50.88	50.69	
2–3 times a week	17.34	19.72	17.41	14.70	
4–6 times a week	8.76	9.13	9.07	8.04	
Daily or more	4.99	4.64	5.62	4.71	
Physical activity (%)					
Vigorous	24.53	33.41	22.68	16.86	<0.01
Moderate	28.42	25.77	30.89	28.67	
Less than moderate	47.05	40.82	46.43	54.46	
BMI (kg/m^2^)	28.79 ± 6.72	26.43 ± 6.21	28.92 ± 6.61	31.20 ± 6.48	<0.01
Smoke (%)	20.60	19.43	20.88	21.57	<0.01
Hypertension (%)	31.95	21.12	32.14	43.49	<0.01
Diabetes (%)	9.07	2.85	6.64	18.38	<0.01
Cholesterol (mg/dl)	194.11 ± 41.21	181.25 ± 35.46	194.95 ± 39.37	207.15 ± 44.54	<0.01
Triglyceride (mg/dl)	155.17 ± 132.15	69.74 ± 19.39	128.24 ± 28.30	276.24 ± 174.11	<0.01
Glucose (mg/dl)	99.81 ± 34.60	87.62 ± 12.30	95.10 ± 18.49	118.01 ± 52.17	<0.01
TyG index	8.71 ± 0.72	7.97 ± 0.31	8.68 ± 0.17	9.55 ± 0.51	<0.01
Nephrolithiasis (%)	9.30	6.98	9.15	11.98	<0.01
Nephrolithiasis recurrence (%)	3.17	1.84	3.27	4.50	<0.01

TyG, triglyceride glucose; GED, general educational development; RIP, ratio of family income to poverty; BMI, body mass index.

^1^BMI was categorized as <25, 25–29.9, and ≥30 kg/m^2^, which corresponded to normal weight, overweight, and obese population, respectively.

### Higher Triglyceride–Glucose Index Is Associated With Higher Likelihood of Kidney Stones

For nephrolithiasis, a positive association between TyG index and nephrolithiasis was observed. In the fully adjusted model (Model 3), this positive association was still stable (OR = 1.12; 95% CI: 1.02–1.22; p = 0.02), indicating that each unit of increased TyG index was associated with 12% increased risk of nephrolithiasis. We also converted TyG index from a continuous variable to a categorical variable (tertiles) to conduct sensitivity analysis. Compared with the lowest TyG index tertile (Tertile 1), a significant 18% increased likelihood of kidney stones was observed in Tertile 3. However, the difference between Tertile 1 and Tertile 2 did not meet statistical significance (OR = 1.04; 95% CI: 0.89–1.21; p = 0.66) **(** **Table 2** **)**.

**Table 2:** Association of TyG with kidney stone and a recurrence of passing kidney stones.

	OR (95% CI), p value		
	Model 1^1^	Model 2^2^	Model 3^3^
	(n = 20,972)	(n = 20,972)	(n = 15,019)
Nephrolithiasis			
TyG index	1.40 (1.32, 1.49) <0.01	1.25 (1.17, 1.34) <0.01	1.12 (1.02, 1.22) 0.02
Categories			
Tertile 1	Reference	Reference	Reference
Tertile 2	1.38 (1.22, 1.56) <0.01	1.15 (1.01, 1.30) 0.04	1.04 (0.89, 1.21) 0.66
Tertile 3	1.84 (1.63, 2.07) <0.01	1.42 (1.26, 1.61) <0.01	1.18 (1.00, 1.38) 0.048
Nephrolithiasis recurrence			
TyG index	1.58 (1.43, 1.75) <0.01	1.44 (1.29, 1.60) <0.01	1.26 (1.08, 1.46) <0.01
Categories			
Tertile 1	Reference	Reference	Reference
Tertile 2	1.76 (1.39, 2.22) <0.01	1.46 (1.16, 1.85) <0.01	1.25 (0.95, 1.65) 0.11
Tertile 3	2.61 (2.09, 3.25) <0.01	2.02 (1.61, 2.53) <0.01	1.59 (1.20, 2.11) <0.01

In sensitivity analysis, TyG was converted from a continuous variable to a categorical variable (tertiles).

OR, odds ratio; 95% CI, 95% confidence interval.

^1^Model 1: No covariates were adjusted.

^2^Model 2: Adjusted for gender, age, and race.

^3^Model 3: Adjusted for gender, age, race, education level, poverty-to-income ratio, marital status, alcohol intake, physical activity, cholesterol, body mass index, smoking status, hypertension, and diabetes.

As for the recurrence of nephrolithiasis, we also observed that increased TyG index was associated with higher odds of nephrolithiasis recurrence (Model 1: OR = 1.58, 95% CI: 1.43–1.75, p < 0.01; Model 2: OR = 1.44, 95% CI: 1.29–1.60, p < 0.01; Model 3: OR = 1.26, 95% CI: 1.08–1.46, p < 0.01). In Model 3 that adjusted for all covariates, our results indicated that each unit of increased TyG index was associated with 26% increased likelihood of nephrolithiasis recurrence. In the sensitivity analysis, the adjusted OR (reference to Tertile 1) was 1.59 (95% CI: 1.20–2.11; p < 0.01) for Tertile 3, suggesting a stable positive relationship between increased TyG index and increased odds of nephrolithiasis recurrence with statistical significance **(** **Table 2** **)**.

**Figure 2:**
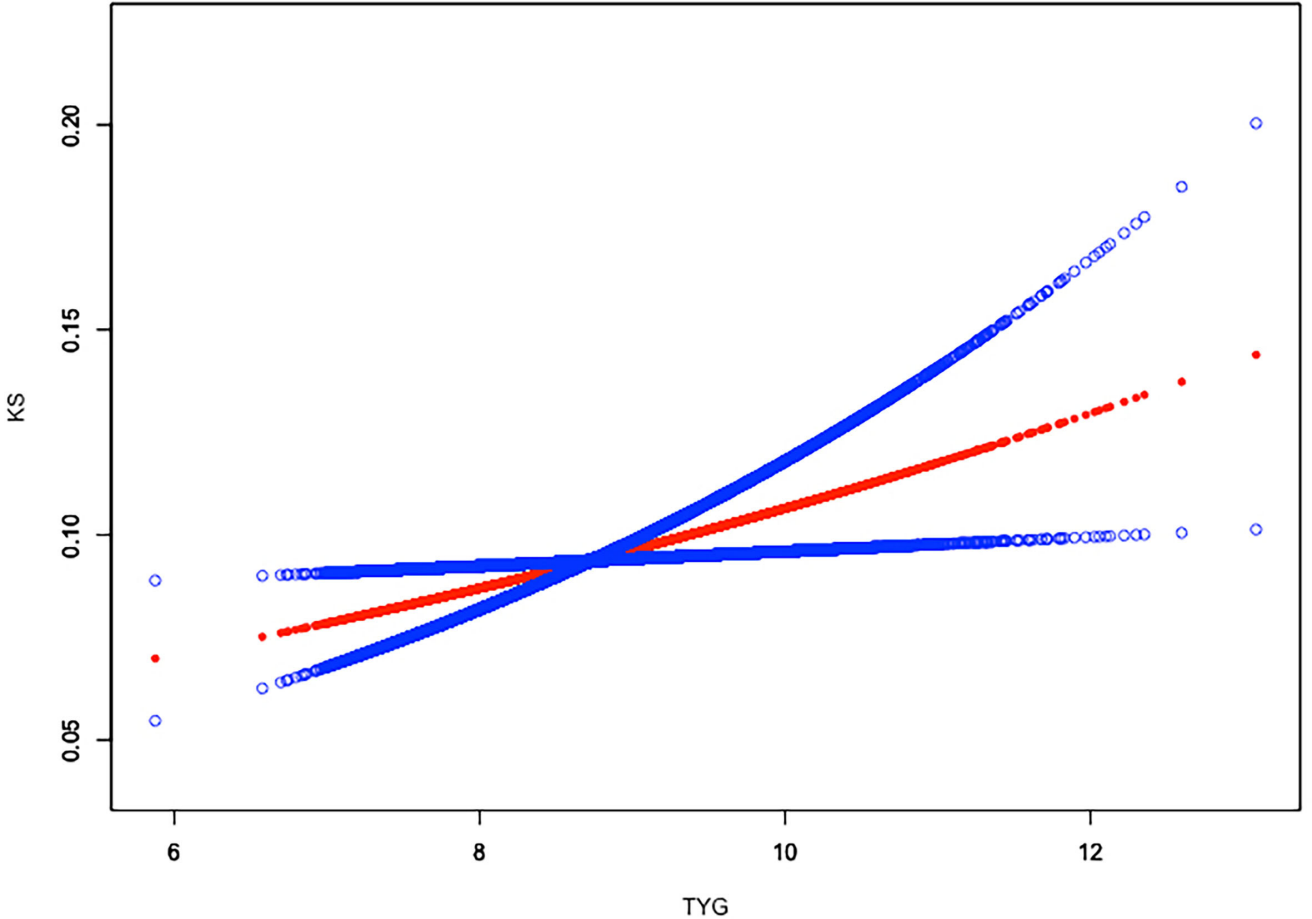
Linear relationship between triglyceride–glucose (TyG) index and kidney stones by the generalized additive model.

**Figure 3:**
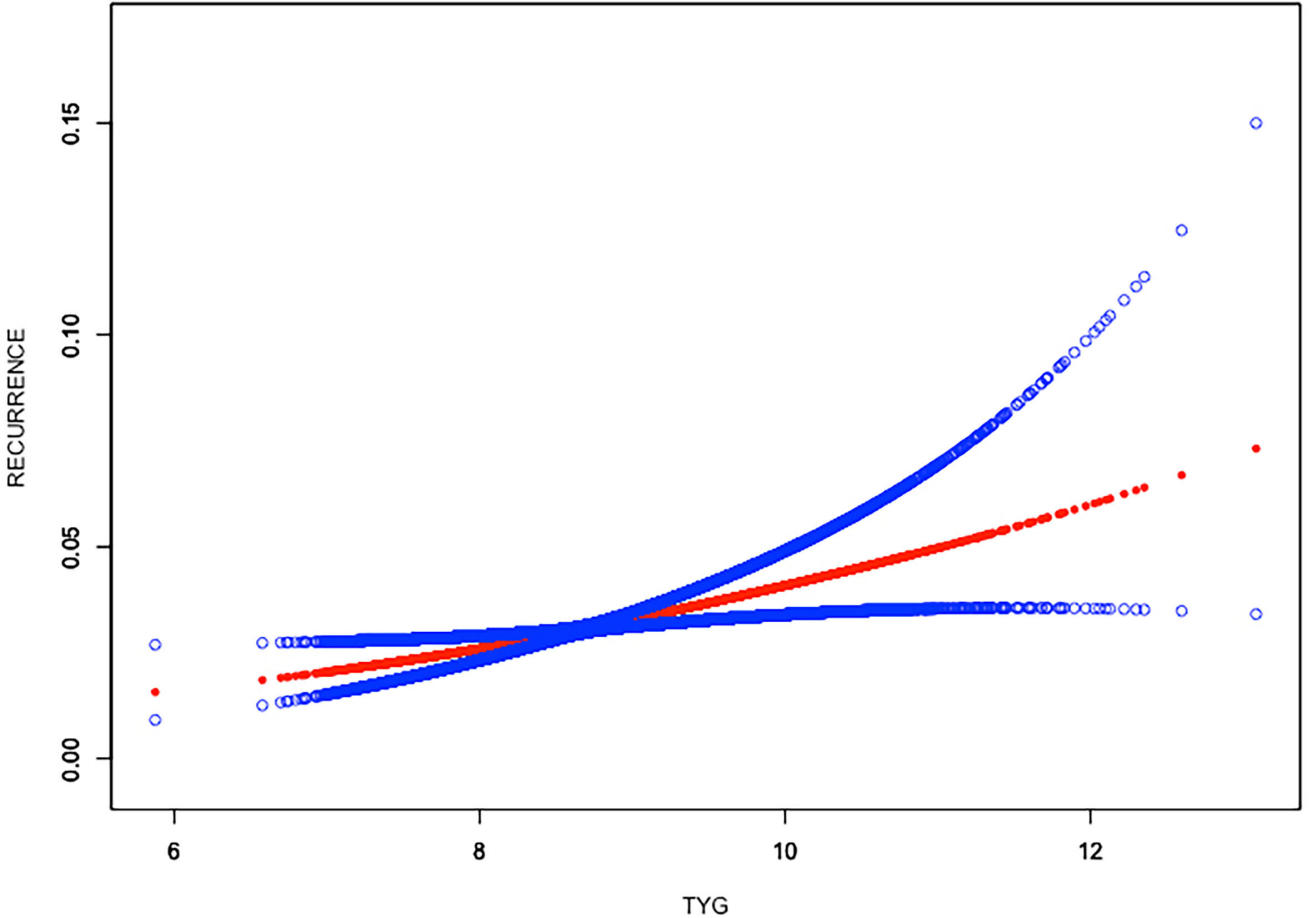
Linear relationship between triglyceride–glucose (TyG) index and the recurrence of kidney stones by the generalized additive model.

Weighted generalized additive models and smooth curve fittings were employed to further explore the nonlinear relationship between TyG index and kidney stones. Our results indicated that there was no nonlinear relationship between TyG index with nephrolithiasis **(** **Figure 2** **)**. Similar result was observed in the association between TyG index and nephrolithiasis recurrence as well (all p > 0.05), indicating that the relationship between TyG index and kidney stones was linear **(** **Figure 3** **)**.

### Subgroup Analysis

Subgroup analysis was performed to evaluate the robustness of association between TyG index and kidney stones. We tested the interactions with gender, age, BMI, hypertension, and diabetes (including both type 1 and type 2 diabetes) as well. However, no correlation with the p for interaction meeting the statistical significance was detected, indicating that there was no dependence on gender, age, BMI, hypertension, and diabetes for this association (all p for interaction >0.05). Our results showed that this positive association of TyG index and kidney stones was similar in populations with different genders, ages, BMIs, and hypertension and diabetes statuses and could be appropriate for various population settings. It was worth noting that we could not further conduct the subgroup analysis stratified by type 1 and type 2 diabetes, since NHANES data did not clarify type 1 or type 2 diabetes in its study design (**Table 3**).

**Table 3:** Subgroup analysis.

DII	Kidney stone		Recurrence of passing kidney stones	
	OR (95% CI), p for trend	p for interaction	OR (95% CI), p for trend	p for interaction
Subgroup analysis stratified by gender				
Male (n = 7,692)	1.09 (0.97, 1.22) 0.14	0.21	1.29 (1.07, 1.56) <0.01	0.87
Female (n = 7,327)	1.20 (1.03, 1.39) 0.02		1.25 (0.97, 1.61) 0.08	
Subgroup analysis stratified by age (years)				
Age <60 (n = 9,708)	1.08 (0.95, 1.22) 0.24	0.47	1.16 (0.95, 1.42) 0.15	0.31
Age ≥60 (n = 5,311)	1.19 (1.03, 1.36) 0.01		1.39 (1.12, 1.74) <0.01	
Subgroup analysis stratified by BMI^1^ (kg/m^2^)				
Normal weight (n = 4,347)	1.11 (0.89, 1.38) 0.37	0.22	1.17 (0.79, 1.72) 0.44	0.76
Overweight (n = 5,034)	1.01 (0.86, 1.18) 0.91		1.21 (0.93, 1.56) 0.15	
Obese (n = 5,548)	1.22 (1.07, 1.40) <0.01		1.33 (1.08, 1.64) <0.01	
Subgroup analysis stratified by hypertension				
Yes (n = 5,355)	1.16 (1.03, 1.32) 0.02	0.74	1.19 (0.96, 1.46) 0.10	0.52
No (n = 9,664)	1.05 (0.93, 1.20) 0.42		1.31 (1.06, 1.61) 0.01	
Subgroup analysis stratified by diabetes				
Yes (n = 1,740)	1.19 (1.00, 1.41) 0.04	0.57	1.18 (1.01, 1.55) 0.04	0.37
No (n = 13,279)	1.09 (0.98, 1.21) 0.11		1.29 (1.08, 1.53) 0.01	

The results of subgroup analysis were adjusted for all covariates except effect modifier.

OR, odds ratio; 95% CI, 95% confidence interval.

^1^BMI was categorized as <25, 25–29.9, and ≥30 kg/m^2^, which corresponded to normal weight, overweight, and obese population, respectively.

## Discussion

In this cross-sectional study that included 20,972 adults, we found that higher TyG index was independently associated with increased likelihood of nephrolithiasis and nephrolithiasis recurrence. This association was similar in subgroups stratified by gender, age, BMI, hypertension, and diabetes status. We postulate that treatment and management of IR at a younger age might be beneficial to improve or alleviate the occurrence and recurrence of kidney stones.

To our knowledge, this is the first study assessing the association between TyG index and kidney stones. Previous studies have reported the relationship of kidney stones with several other clinicopathological factors. Maddahi et al. (17) revealed that diet with a higher dietary inflammatory index (DII) could increase the odds of kidney stone formation in stone former men. Zhang et al. (18) also reported that increased intake of pro-inflammatory diet was correlated with higher odds of kidney stone incidence and recurrence. DII scores serve as a tool to evaluate the effect of diet on inflammatory potential. Anti-inflammatory dietary patterns, such as DASH diet and Mediterranean diet, could reduce the level of systemic inflammation (19, 20). Some anti-inflammatory metabolites derived from dietary components, such as short-chain fatty acids and tryptophan and tyrosine metabolites, also play a role in the regulation of inflammation. Tryptamine could reduce fatty acid- and LPS-stimulated production of pro-inflammatory cytokines in macrophages and inhibited the migration of cells toward a chemokine in mice (21). Supplementation with desaminotyrosine, a gut microbiota-derived anti-inflammatory metabolite, was proven to attenuate dextran sodium sulfate-induced mucosal inflammation in a type I interferon signal-dependent manner, thus modulating local and systemic immune homeostasis (22). Commensal gut microflora and dietary fiber also regulated inflammation status through inter-organ signaling, thus playing pathophysiologic roles in obesity, diabetes, and dyslipidemia (23, 24). In contrast, higher-DII diets could enhance the systemic inflammation level and resulted in hypercalciuria, hyperuricosuria, and hypocitraturia, thus leading to the formation of kidney stones. Cohen et al. (25) demonstrated a protective effect of statin intake for the formation of kidney stones. Although the certain mechanism was still unclear, there were signs that anti-inflammatory and antioxidant properties of statins may reduce the occurrence of kidney stones. Increased inflammatory status provided conditions for the formation and deposition of renal tubular crystals, which has been confirmed in a mouse model (26). In addition, statin treatment could lower the inflammatory level, then inhibited the renal crystal retention (27). Inflammation was reported to be positively associated with IR as well (28, 29). At the molecular level, the transition of macrophages from a state of alternative M2 activation maintained by signal transducer and activator of transcription (STAT6) and peroxisome proliferators-activated receptors (PPAR) to a state of classic M1 activation driven by Nuclear Factor-KappaB (NF-KappaB), activator protein-1 (AP1), and other signal-dependent transcription factors that play a key role in innate immunity promotes IR (30, 31). Semins et al. (32) found that the obese population had a higher risk of kidney stone, while as the degree of obesity stratified by BMI increased, the risk stabilized. Previous studies exploring the effect of body size on urine chemistry demonstrated that increasing BMI could enhance the lithogenic risk factors such as decreased urinary volume and citrate concentrations (33). Chronic inflammation in adipose tissue was considered to be a key risk factor for IR and type 2 diabetes in obese individuals. Obesity-induced adipose tissue expansion provides a number of internal signals (such as adipocyte death, hypoxia, and mechanical stress) that could initiate an inflammatory response (34). Since inflammation was associated with IR and kidney stones and TyG index has been regarded as a reliable indicator of IR, a positive association between TyG index and kidney stones could be speculated.

The mechanism underlying the association between TyG index and kidney stones is not clear. A possible explanation to support our results might be that the greater IR represented by higher TyG index could decrease the excretion of urinary citrate (14). Increased level of plasma free fatty acids could be detected in IR patients, which then got into the proximal tubule cells and interfered with the utilization of glutamine, resulting in a reduction in ammoniagenesis (35). Also, it has been proven that insulin could stimulate renal ammonium production from L-glutamine *in vitro*, representing direct damage of IR to ammoniagenesis (36). In addition, insulin could directly stimulate the Na^+^/H^+^ exchangers of proximal renal tubule that play a key role in transporting or ionic trapping of ammonium (37). In summary, the impaired ability of excreting ammonia in patients with IR could lead to hyperacid urine, which is a main risk factor for the formation of uric acid stones. IR could also increase the uptake of citrate in renal tubules and reduce the urinary citrate, which is a main risk factor for the formation of calcium stones. It has been reported that higher IR level also increased urinary calcium excretion, although the mechanism still remains unclear (38). The fact that the TyG index is positively correlated with the IR level may explain the reason why higher TyG index is associated with increased risk of nephrolithiasis.

Previous epidemiological studies demonstrated that obesity, hypertension, and diabetes were risk factors for nephrolithiasis. Taylor et al. (39) revealed that obesity and weight gain aggravated the risk of kidney stone formation, predominantly in females. One study found that hypertension in middle-aged white men could serve as a marked predictor for nephrolithiasis, which may be associated with greater and sustained urinary calcium losses in patients with hypertension (40). Diabetes was also associated with the prevalence of kidney stone disease, and there existed a positive relation between the severity of type 2 diabetes mellitus, which could be explained by the effects of IR on urinary pH and renal transport of ammonium and calcium (41). According to our results of subgroup analysis, the positive association was stable in subgroups stratified by BMI, diabetes, and hypertension, which is consistent with the studies before. In addition, we did not detect any dependence on gender, age, BMI, hypertension, and diabetes for this association (all p for interaction >0.05), suggesting that this positive association may be appropriate for different population settings.

Our study has several strengths. Firstly, our study was based on the data from NHANES and the analyses was conducted considering the appropriate NHANES sample weights. Secondly, we adjusted confounding covariates to ensure our results are reliable and could be applied to a wider range of individuals. However, limitations in our study cannot be ignored. Firstly, the diagnosis of kidney stone was based on personal interview; the recall bias was inevitable. Secondly, since NHANES data did not clarify type 1 or type 2 diabetes in its study design, we could not further evaluate this association for subgroup stratified by type 1 and type 2 diabetes. In addition, since the missing data of covariables were missed randomly and the sample size was large enough to draw a conclusion, we did not use multiple imputation to deal with the missing data, thus it may influence the accuracy. Due to the cross-sectional study design, we could not obtain a causal relationship between TyG and kidney stones.

## Conclusion

Higher TyG index was associated with an increased likelihood of kidney stone incidence and recurrence. We postulate that treatment and management of IR at a younger age may improve or alleviate the occurrence and recurrence of kidney stones. However, further large-scale prospective studies are still needed to clarify the precise causality of this relationship.

## Data Availability Statement

Publicly available datasets were analyzed in this study. These data can be found here: https://www.cdc.gov/nchs/nhanes/.

## Ethics Statement

The studies involving human participants were reviewed and approved by The NCHS Research Ethics Review Board. The patients/participants provided their written informed consent to participate in this study.

## Author Contributions

ZQ: data analysis, software, and writing—original draft. JZ: formal analysis and writing—original draft. JG: methodology and software. KC: data analysis. RL: software and funding acquisition. BS: conceptualization, funding acquisition, and writing—reviewing and editing. All authors contributed to the article and approved the submitted version.

## Funding

This work was supported by the National Natural Science Foundation of China (Grant No. 82000702), the Science and Technology Achievement Transformation Fund of West China Hospital of Sichuan University (Grant No. CGZH19006), the 1.3.5 project for disciplines of excellence from West China Hospital of Sichuan University (Grant No. ZYJC21010), National Clinical Research Center for Geriatrics, West China Hospital, Sichuan University (Grant No. Z2018B10), and Med+ Biomaterial Institute of West China Hospital/West China School of Medicine of Sichuan University (Grant No. ZYME20001).

## Conflict of Interest

The authors declare that the research was conducted in the absence of any commercial or financial relationships that could be construed as a potential conflict of interest.

## Publisher’s Note

All claims expressed in this article are solely those of the authors and do not necessarily represent those of their affiliated organizations, or those of the publisher, the editors and the reviewers. Any product that may be evaluated in this article, or claim that may be made by its manufacturer, is not guaranteed or endorsed by the publisher.
